# Supplementary material for: Dynamic Factor Analysis for Sparse and Irregular Longitudinal Data: An Application to Metabolite Measurements in a COVID‐19 Study
Source: Stat Med. 2026 Mar 16;45(6-7):e70499. doi: 10.1002/sim.70499 (PMC12992701; doi:10.1002/sim.70499)
Supplement: Supplementary file 1 — Data S1. Supporting information. [file SIM-45-0-s001.pdf]

Supporting Information for Dynamic factor analysis for sparse and irregular longitudinal data: an application to metabolite measurements in a COVID-19 study by Jiachen Cai, Robert J. B. Goudie, Brian D. M. Tom

A. Mathematical derivations

A.1 Full conditionals of the Gibbs sampler for the proposed model

Throughout the following derivation, "$\circ$" denotes element-wise multiplication, "$-$" denotes observed data and all parameters in the model other than the parameter under derivation, "$||\mathbf{z}||^2$" denotes the sum of squares of each element of the vector $\mathbf{z}$, "diag($\mathbf{z}$)" denotes a diagonal matrix with the vector $\mathbf{z}$ as its main diagonal elements, "MVN $(\mathbf{z}; \boldsymbol{\mu}_{\mathbf{z}}, \Sigma_{\mathbf{z}})$" denotes the probability density function of a multivariate normal distribution with mean $\boldsymbol{\mu}_{\mathbf{z}}$, and variance $\Sigma_{\mathbf{z}}$, and similar interpretations apply to other distributions. "$\mathbf{z}^T$" denotes the transpose of the vector or matrix $\mathbf{z}$, and "pos" is short for 'posterior probability'.

- Full conditional for the latent factors $\text{vec}({\mathbf{Y}_{i,\text{aug}}}^T), i = 1, \ldots, n$

$$f(\text{vec}({\mathbf{Y}_{i,\text{aug}}}^T) \mid -) = f(\text{vec}(\mathbf{Y}_i^T) \mid -) \cdot f(\text{vec}(\mathbf{Y}_{i,\text{add}}^T) \mid \text{vec}(\mathbf{Y}_i^T), \Sigma_{\mathbf{Y}_{\text{aug}}}(\mathbf{\Theta}, \mathbf{t}))$$

We first sample $\text{vec}(\mathbf{Y}_i^T)$ according to:

$$\begin{aligned} f(\text{vec}(\mathbf{Y}_i^T) \mid -) &\propto \text{MVN}\left(\text{vec}(\mathbf{X}_i^T);\ \text{vec}(\mathbf{M}_i^T) + \mathbf{L}_i^* \text{vec}(\mathbf{Y}_i^T),\ \Sigma_{\mathbf{X}_i}\right) \cdot \text{MVN}\left(\text{vec}(\mathbf{Y}_i^T);\ \mathbf{0},\ \Sigma_{\mathbf{Y}_i}(\mathbf{\Theta}, \mathbf{t}_i)\right) \\ &= \text{MVN}\left(\mu^{\text{pos}}_{\mathbf{Y}_{i,}},\ \Sigma^{\text{pos}}_{\mathbf{Y}_{i,}}\right), \end{aligned}$$

with

$$\begin{aligned} \Sigma^{\text{pos}}_{\mathbf{Y}_{i,}} &= [\mathbf{L}_i^{*T} \Sigma_{\mathbf{X}_i}^{-1} \mathbf{L}_i^* + \Sigma_{\mathbf{Y}_i}(\mathbf{\Theta}, \mathbf{t}_i)^{-1}]^{-1} \\ \mu^{\text{pos}}_{\mathbf{Y}_{i,}} &= \Sigma^{\text{pos}}_{\mathbf{Y}_{i,}}(\mathbf{L}_i^{*T} \Sigma_{\mathbf{X}_i}^{-1}(\text{vec}(\mathbf{X}_i^T) - \text{vec}(\mathbf{M}_i^T))). \end{aligned}$$

Then we sample $\text{vec}(\mathbf{Y}_{i,\text{add}}^T)$ from $f(\text{vec}(\mathbf{Y}_{i,\text{add}}^T) | \text{vec}(\mathbf{Y}_i^T),\ \Sigma_{\mathbf{Y}_{\text{aug}}}(\mathbf{\Theta}, \mathbf{t}))$, a MVN distribution due to the property of the MOGP model (Shi & Choi, 2011).

In the above equations:

- $\Sigma_{\mathbf{Y}_{\text{aug}}}(\mathbf{\Theta}, \mathbf{t})$ is the covariance matrix of factor scores at full time $\mathbf{t}$, and $\Sigma_{\mathbf{Y}_i}(\mathbf{\Theta}, \mathbf{t}_i)$ is a sub-matrix of it (at subject-specific time points);
- $\mathbf{L}_i^*$ is constructed using components of the factor loading matrix $\mathbf{L}$: $\mathbf{L}_i^* = (\mathbf{L}_1^*, ..., \mathbf{L}_p^*)^T \in \mathbb{R}^{pq_i \times kq_i}$, where $(\mathbf{L}_g^*)^T = (\text{diag}(l_{g1})_{q_i \times q_i}, ..., \text{diag}(l_{gk})_{q_i \times q_i}) \in \mathbb{R}^{q_i \times kq_i}$;
- $\Sigma_{\mathbf{X}_i} = \text{diag}((\phi_1^2)_{\times q_i}, ..., (\phi_p^2)_{\times q_i}) \in \mathbb{R}^{pq_i \times pq_i}$, where $(\phi_a^2)_{\times q_i}$ represents a q_i-dimensional row vector consisting of the scalar ϕ_a^2.

Note that when coding the algorithm, there is no need to directly create the $pq_i \times pq_i$ diagonal matrix $\Sigma_{\mathbf{X}_i}^{-1}$ as the memory will be exhausted. To calculate the term $\mathbf{L}_i^{*T} \Sigma_{\mathbf{X}_i}^{-1}$, we can use the property of multiplication of a diagonal matrix: post-multiplying a diagonal matrix is equivalent to multiplying each column of the first matrix by corresponding elements in the diagonal matrix.

- Full conditional for the binary matrix $\mathbf{Z}$

  Let $\mathbf{Z}_{g\cdot} = (Z_{g1}, ..., Z_{gk})$ denote the gth row of the matrix $\mathbf{Z}$, $g = 1, ..., p$; then,

$$f(\mathbf{Z}_{g\cdot}|-) \propto \prod_{i=1}^{n} \text{MVN}\left(\mathbf{x}_{ig};\ \boldsymbol{\mu}_{ig} + (\mathbf{A}_{g\cdot} \circ \mathbf{Z}_{g\cdot})\mathbf{Y}_i,\ \text{diag}(\phi_g^2,\ q_i)\right) \cdot \prod_{a=1}^{k} \text{Bernoulli}(Z_{ga}; \pi_a),$$

  We calculate the posterior probability under 2^k possible values of $\mathbf{Z}_{g\cdot}$ based on the above formula, then sample with corresponding probability.

- Full conditional for the regression coefficient matrix $\mathbf{A}$

  Let $\mathbf{A}_{g\cdot} = (A_{g1}, ..., A_{gk})$ denote the gth row of the matrix $\mathbf{A}$, $g = 1, ..., p$; then,

$$\begin{aligned} f(\mathbf{A}_{g\cdot}|-) &\propto \prod_{i=1}^{n} \text{MVN}\left(\mathbf{x}_{ig};\ \boldsymbol{\mu}_{ig} + (\mathbf{A}_{g\cdot} \circ \mathbf{Z}_{g\cdot})\mathbf{Y}_i,\ \text{diag}(\phi_g^2,\ q_i)\right) \cdot \text{MVN}\left(\mathbf{A}_{g\cdot};\ \mathbf{0},\ \text{diag}(\boldsymbol{\rho}^2)\right) \\ &= \text{MVN}\left(\mathbf{A}_{g\cdot};\ \mu^{\text{pos}}_{\mathbf{A}_{g\cdot}},\ \Sigma^{\text{pos}}_{\mathbf{A}_{g\cdot}}\right), \end{aligned}$$

  where

$$\begin{aligned} \Sigma^{\text{pos}}_{\mathbf{A}_{g\cdot}} &= \left(\frac{\text{diag}(\mathbf{Z}_{g\cdot})(\sum_{i=1}^{n} \mathbf{Y}_i{}^T \mathbf{Y}_i)\text{diag}(\mathbf{Z}_{g\cdot})}{\phi_g^2} + \text{diag}(\frac{1}{\boldsymbol{\rho}^{\mathbf{2}}})\right)^{-1} \\ \mu^{\text{pos}}_{\mathbf{A}_{g\cdot}} &= \frac{\Sigma_{\mathbf{A}_{g\cdot}}(\text{diag}(\mathbf{Z}_{g\cdot}) \sum_{i=1}^{n} \mathbf{Y}_i(\mathbf{x}_{ig} - \boldsymbol{\mu}_{ig}))}{\phi_g^2}. \end{aligned}$$

  and $\boldsymbol{\rho}^{\mathbf{2}} = (\rho_1^2, ..., \rho_a^2)$.

- Full conditional for the intercept $\mu_{ig}, i = 1, ..., n; g = 1, ..., p$

$$\begin{aligned} f(\mu_{ig}|-) &\propto \prod_{j=1}^{q_i} \text{N}(x_{ijg}; \mu_{ig} + \sum_{a=1}^{k} l_{ga} y_{ija}, \phi_g^2) \cdot \text{N}(\mu_{ig}; \mu_g, \sigma_g^2) \\ &= \text{N}(\mu_{ig}; \mu_{ig}^{\text{pos}},\ \sigma_{ig}^{2,\text{pos}}), \end{aligned}$$

  where

$$\begin{aligned} \sigma_{ig}^{2,\text{pos}} &= (\frac{1}{\sigma_g^2} + \frac{q_i}{\phi_g^2})^{-1} \\ \mu_{ig}^{\text{pos}} &= \left(\frac{\mu_g}{\sigma_g^2} + \frac{\sum_{j=1}^{q_i}(x_{ijg} - \sum_{a=1}^{k} l_{ga} y_{ija})}{\phi_g^2}\right) \cdot \sigma_{ig}^{2,\text{pos}} \end{aligned}$$

- Full conditional for π_a, $a = 1, ..., k$

$$\begin{aligned} f(\pi_a|-) &\propto \prod_{a=1}^{k} \text{Bernoulli}(Z_{ga}; \pi_a) \cdot \text{Beta}(\pi_a; c_0, d_0) \\ &= \text{Beta}\left(c_0 + \sum_{g=1}^{p} Z_{ga},\ d_0 + \sum_{g=1}^{p}(1 - Z_{ga})\right) \end{aligned}$$

- Full conditional for $\rho_a^2,\ a = 1, ..., k$

$$f(\rho_a^2|-) \propto \prod_{g=1}^{p} \mathrm{N}(A_{ga}; 0, \rho_a^2) \cdot \text{Inverse-Gamma}(\rho_a^2; c_1, d_1)$$
$$= \text{Inverse-Gamma}\left(c_1 + \frac{p}{2},\ d_1 + \frac{1}{2}\sum_{g=1}^{p} A_{ga}^2\right)$$

- Full conditional for $\sigma_g^2,\ g = 1, ..., p$

$$f(\sigma_g^2|-) \propto \prod_{i=1}^{n} \mathrm{N}(\mu_{ig}; \mu_g, \sigma_g^2) \cdot \text{Inverse-Gamma}(\sigma_g^2; c_2, d_2)$$
$$= \text{Inverse-Gamma}\left(c_2 + \frac{1}{2}n,\ d_2 + \frac{1}{2}\sum_{i=1}^{n} (\mu_{ig} - \mu_g)^2\right)$$

- Full conditional for $\phi_g^2,\ g = 1, ..., p$

$$f(\phi_g^2|-) \propto \prod_{i=1}^{n} \mathrm{MVN}(\mathbf{x}_{ig};\ \boldsymbol{\mu}_{ig} + (\mathbf{A}_{g\cdot} \circ \mathbf{Z}_{g\cdot})\mathbf{Y}_i,\ \mathrm{diag}(\phi_g^2,\ q_i)) \cdot \text{Inverse-Gamma}(\phi_g^2; c_3, d_3)$$
$$= \text{Inverse-Gamma}\left(c_3 + \frac{1}{2}\sum_{i=1}^{n} q_i,\ d_3 + \frac{1}{2}\sum_{i=1}^{n} ||\mathbf{x}_{ig} - \boldsymbol{\mu}_{ig} - (\mathbf{A}_{g\cdot} \circ \mathbf{Z}_{g\cdot})\mathbf{Y}_i||^2\right)$$

- Full conditional for predictions of biomarker expression (only implemented when recovering the full trajectories of biomarkers, during the Gibbs-after-StEM stage)

  Suppose that $\mathbf{X}_i^{\text{new}}, \mathbf{Y}_i^{\text{new}}$ represent predicted biomarker expression and factor expression of the ith individual at new time points, respectively. The posterior predictive distribution under StEM-algorithm-returned $\widehat{\boldsymbol{\Theta}}^{\text{MLE}}$ can be expressed as

$$f(\mathbf{X}_i^{\text{new}}|\ \widehat{\boldsymbol{\Theta}}^{\text{MLE}}, \boldsymbol{\Omega}_{\text{aug}}) = \int f(\mathbf{X}_i^{\text{new}}, \mathbf{Y}_i^{\text{new}}|\ \widehat{\boldsymbol{\Theta}}^{\text{MLE}}, \boldsymbol{\Omega}_{\text{aug}}) d\mathbf{Y}_i^{\text{new}}$$
$$= \int f(\mathbf{X}_i^{\text{new}}|\mathbf{Y}_i^{\text{new}}, \boldsymbol{\Omega}_{\text{aug}}) \cdot f(\mathbf{Y}_i^{\text{new}}|\widehat{\boldsymbol{\Theta}}^{\text{MLE}}, \mathbf{Y}_i) d\mathbf{Y}_i^{\text{new}},$$

  where the first term of the integrand is a MVN because of the assumed factor model, and the second term is also a MVN because of the assumed DGP model on latent factor trajectories (Shi & Choi, 2011). Therefore, once a sample of parameters $\boldsymbol{\Omega}_{\text{aug}}$ is generated, the sample of $\mathbf{Y}_i^{\text{new}}$ can be generated from $f(\mathbf{Y}_i^{\text{new}}|\ \widehat{\boldsymbol{\Theta}}^{\text{MLE}}, \mathbf{Y}_i)$, then the sample of $\mathbf{X}_i^{\text{new}}$ can be generated from $f(\mathbf{X}_i^{\text{new}}|\ \mathbf{Y}_i^{\text{new}}, \boldsymbol{\Omega})$.

A.2 Identifiability of the covariance matrix for latent factors

To facilitate illustrating the identifiability issue, we first re-express the proposed model in Section 2.3 as,

$$\begin{aligned} \mathrm{vec}(\mathbf{X}_i^T) &= \mathrm{vec}(\mathbf{M}_i^T) + \mathbf{L}_i^* \,\mathrm{vec}(\mathbf{Y}_i^T) + \mathrm{vec}(\mathbf{E}_i^T), \\ \mathrm{vec}({\mathbf{Y}_i}^T) &\sim \mathrm{MVN}(\mathbf{0}, \Sigma_{\mathbf{Y}_i}), \\ \mathrm{vec}(\mathbf{E}_i^T) &\sim \mathrm{MVN}(\mathbf{0}, \Sigma_{\mathbf{X}_i}), \end{aligned} \tag{1}$$

where $\mathrm{vec}(\mathbf{X}_i^T)$, $\mathrm{vec}(\mathbf{M}_i^T)$, and $\mathrm{vec}(\mathbf{E}_i^T)$ are vectorized from matrices $\mathbf{X}_i^T$, $\mathbf{M}_i^T$, and $\mathbf{E}_i^T$, respectively. To make the above equations hold, $\mathbf{L}_i^*$ and $\Sigma_{\mathbf{X}_i}$ are constructed using components of $\mathbf{L}$ and $\boldsymbol{\phi}$, respectively. Specifically,

- $\mathbf{L}_i^* = (\mathbf{L}_1^*, ..., \mathbf{L}_p^*)^T \in \mathbb{R}^{pq_i \times kq_i}$, where $(\mathbf{L}_g^*)^T = (\mathrm{diag}(l_{g1})_{q_i \times q_i}, ..., \mathrm{diag}(l_{gk})_{q_i \times q_i}) \in \mathbb{R}^{q_i \times kq_i}$;
- $\Sigma_{\mathbf{X}_i} = \mathrm{diag}((\phi_1^2)_{1\times q_i}, ..., (\phi_p^2)_{1\times q_i}) \in \mathbb{R}^{pq_i \times pq_i}$, where $(\phi_a^2)_{1\times q_i}$ represents a q_i-dimensional row vector consisting of the scalar ϕ_a^2.

The distribution of $\mathrm{vec}(\mathbf{X}_i^T)$ after integrating out $\mathrm{vec}(\mathbf{Y}_i^T)$, is

$$\mathrm{vec}(\mathbf{X}_i^T) \mid \mathrm{vec}(\mathbf{M}_i^T), \mathbf{L}_i^*, \Sigma_{\mathbf{X}_i}, \Sigma_{\mathbf{Y}} \sim \mathrm{MVN}(\mathrm{vec}(\mathbf{M}_i^T), \mathbf{L}_i^* \Sigma_{\mathbf{Y}_i} (\mathbf{L}_i^*)^T + \Sigma_{\mathbf{X}_i}), \tag{2}$$

where $\Sigma_{\mathbf{Y}_i}$ is a sub-matrix of $\Sigma_{\mathbf{Y}}$ that characterizes the covariance structure for $\mathrm{vec}(\mathbf{Y}_i^T)$.

As noted, there is an identifiability issue with the covariance matrix $\Sigma_{\mathbf{Y}_i}$. This issue arises from the invariance of the covariance $\mathbf{L}_i^* \Sigma_{\mathbf{Y}_i} (\mathbf{L}_i^*)^T + \Sigma_{\mathbf{X}_i}$ in Equation 2. The uniqueness of $\Sigma_{\mathbf{X}_i}$ has been ensured in previous research (Ledermann, 1937; Bekker & ten Berge, 1997; Conti et al., 2014; Papastamoulis & Ntzoufras, 2022); given its identifiability, we are concerned with identifiability of $\Sigma_{\mathbf{Y}_i}$. Non-identifiability is present because for any non-singular transformation matrix $\mathbf{D} \in \mathbb{R}^{kq_i \times kq_i}$, the expression $\mathbf{L}_i^* \Sigma_{\mathbf{Y}_i} (\mathbf{L}_i^*)^T + \Sigma_{\mathbf{X}_i}$ are equal under these two sets of estimators for $\mathbf{L}_i^*$, $\mathrm{vec}(\mathbf{Y}_i^T)$ and $\Sigma_{\mathbf{Y}_i}$: the first estimator is $\{\widehat{\mathbf{L}_i^*}, \widehat{\mathrm{vec}(\mathbf{Y}_i^T)}, \widehat{\Sigma}_{\mathbf{Y}_i}\}$ and the second estimator is $\{\widehat{\mathbf{L}_i^*}\mathbf{D}, \mathbf{D}^{-1}\widehat{\mathrm{vec}(\mathbf{Y}_i^T)}, \mathbf{D}^{-1}\widehat{\Sigma}_{\mathbf{Y}_i}(\mathbf{D}^{-1})^T\}$.

To address this issue, we place a constraint on $\Sigma_{\mathbf{Y}}$ that requires its main diagonal element to be 1. In other words, the covariance matrix of latent factors $\Sigma_{\mathbf{Y}}$ is forced to be a correlation

matrix by assuming the variance of factors to be 1. This restriction has also been used in Conti et al., 2014, with multiple purposes: first, it ensures the uniqueness of $\Sigma_{\mathbf{Y}}$; in turn, it helps set the scale of $\text{vec}(\mathbf{Y}_i^T)$ and consequently helps set the scale of $\mathbf{L}_i^*$.

B. COVID-19 data application

B.1 Reference grid approach

The reference grid approach assumes the reference times as $\mathbf{t}_{\text{ref}} = \{0, 7, 14, ..., 49\}$, and replaces the actual observed time t_{ij} (the jth time point of the ith subject) with the time from the reference grid, denoted as t_{ij}^{new} below. Specifically, the replacement involves the following steps:

1. First, we fixed the first observed time point t_{i1} as the value taken from the reference grid t_{ref} (whichever is closet to t_{i1});

2. Second, for each of the remaining observed time points (i.e., t_{ij}, where $j = 2, ..., q_i$): we calculated the corresponding time after transformation t_{ij}^{new} using the formula $t_{ij}^{\text{new}} = t_{i(j-1)}^{\text{new}} + (t_{ij} - t_{i(j-1)})$, aiming to keep the distance between adjacent times unchanged. If t_{ij}^{new} belongs to the reference grid t_{ref}, keep it; if not, set t_{ij}^{new} as the time in t_{ref} that is closest to it.

Supplementary Figure 1 shows subject-specific observed times before and after such transformation, taking the measurement of a specific biomarker (quinolic acid) as an example. The intra-class correlation coefficient (ICC) of distances between before- and after-transformation times is 0.908, where class corresponds to individual. This number suggests a high correlation of distances within subjects, which reflects that before- and after-translation lines are roughly parallel to each other.

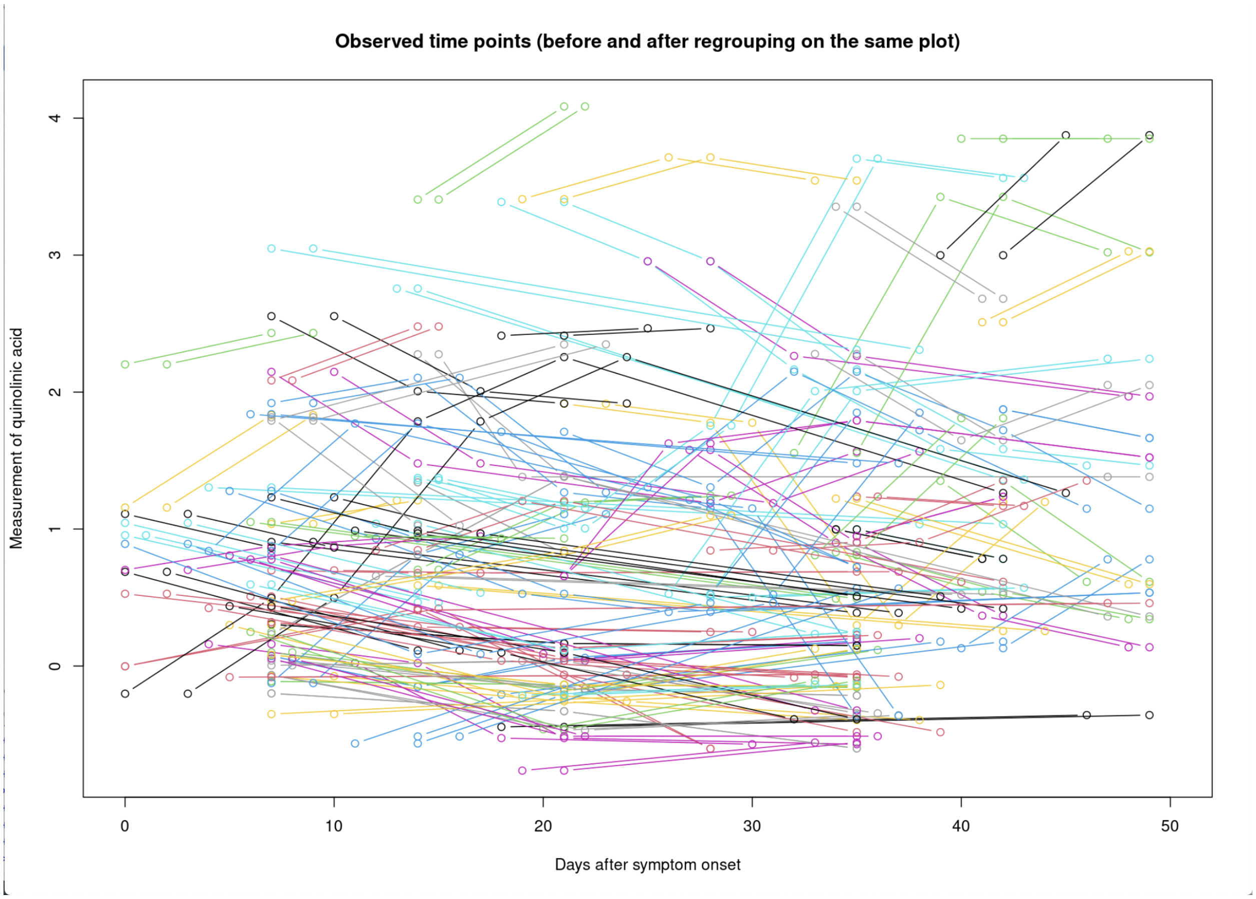

Supplementary Figure 1: Lines connect subject-specific observed times (before- and after-time transformation displayed alongside each other). Points denote the measurement of quinolinic acid at observation times. Note that all metabolites are measured at same times, we choose quinolinic acid as an example to draw the plots.

We compare results using the exact method with $q = 50$ and the approximation method with $q = 8$ (both via our method). Taking the number of latent factors $k = 2$ as an example, Supplementary Tables 1-2 show estimated factor loadings, and Supplementary Figure 2-3 display estimated factor trajectories. Correspondence between metabolite indexes and metabolite names is displayed in Supplementary Table 3.

Both models led to similar results, which is as expected due to the reason described in Section 5.2 of the main manuscript (i.e., the specific design of the COVID-19 study). Note that the individual corresponding to the outlier trajectory with extremely high values (around 10 on day 48) has the largest BMI (37.46) among all patients in the study, which may explain the unusual pathway activity detected by our approach.

Supplementary Table 1: Comparison of estimated loadings on factor 1, between the exact method with $q = 50$ and the approximation method with $q = 8$. The displayed estimate is the median of the posterior samples. Supplementary Table 3 displays metabolite names corresponding to metabolite indexes.

Metabolite index	Exact method with $q = 50$	Approximation method with $q = 8$
1	-0.03	-0.04
2	0.00	0.00
3	0.00	0.00
4	0.00	0.00
5	0.09	0.10
6	0.00	0.00
7	0.00	0.00
8	0.00	0.00
9	0.00	0.00
10	0.00	0.00
11	-0.11	-0.12
12	-0.11	-0.12
13	-0.15	-0.17
14	-0.48	-0.53
15	-0.33	-0.37
16	-0.39	-0.43
17	-0.41	-0.45
18	-0.33	-0.36
19	-0.29	-0.32
20	-0.29	-0.32
21	-0.31	-0.34
22	-0.32	-0.35
23	-0.33	-0.36
24	-0.28	-0.31

25	-0.49	-0.54
26	-0.25	-0.27
27	-0.41	-0.45
28	-0.39	-0.42
29	-0.24	-0.26
30	-0.42	-0.46
31	-0.38	-0.41
32	-0.22	-0.24
33	-0.39	-0.42
34	-0.39	-0.43
35	-0.31	-0.34

Supplementary Table 2: Comparison of estimated loadings on factor 2, between the exact method with $q = 50$ and the approximation method with $q = 8$. The displayed estimate is the median of the posterior samples. Supplementary Table 3 displays metabolite names corresponding to metabolite indexes.

Metabolite index	Exact method with $q = 50$	Approximation method with $q = 8$
1	0.63	0.67
2	0.80	0.86
3	0.50	0.54
4	0.00	0.00
5	0.89	0.95
6	0.64	0.69
7	0.79	0.85
8	0.41	0.44
9	1.22	1.29
10	0.00	0.00
11	-0.17	-0.18
12	0.43	0.45
13	0.24	0.26
14	-0.08	-0.09
15	0.00	0.00
16	0.00	0.00
17	-0.03	-0.02
18	0.00	0.00
19	0.00	0.00
20	0.00	0.00
21	0.00	0.00
22	-0.06	-0.06
23	0.08	0.09
24	0.05	0.06

25	0.00	0.00
26	0.07	0.08
27	0.00	0.00
28	-0.12	-0.13
29	-0.25	-0.28
30	0.00	0.00
31	0.00	0.00
32	0.00	0.00
33	0.04	0.05
34	0.00	0.00
35	0.00	0.00

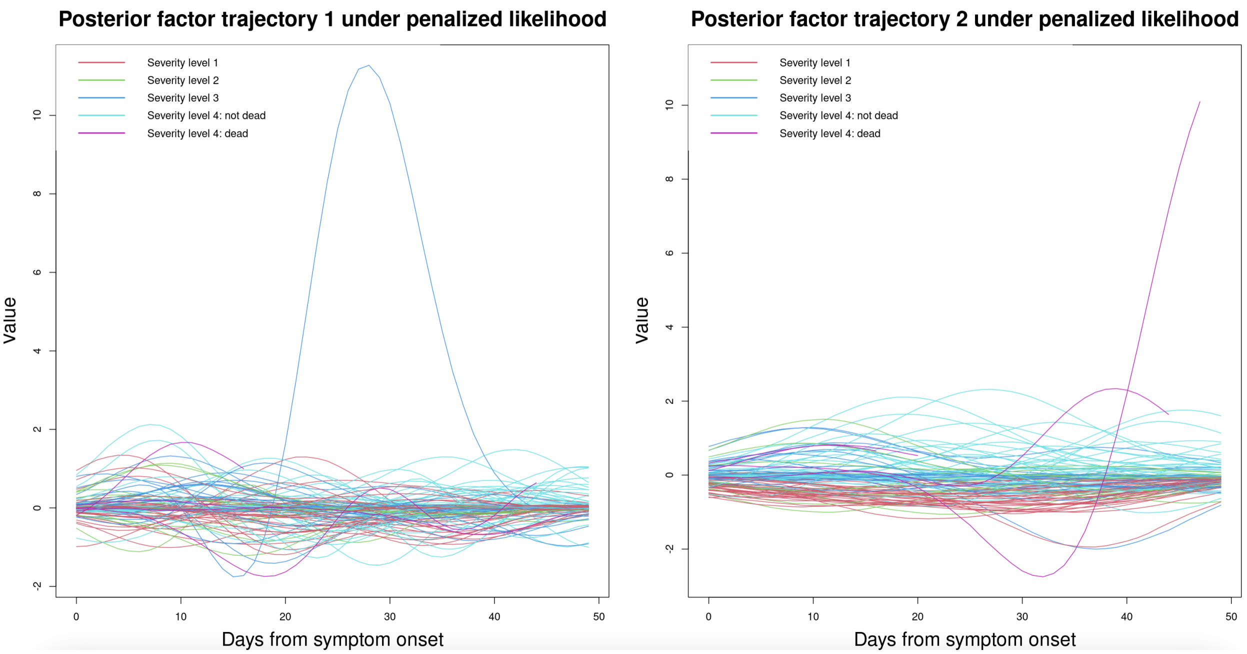

Supplementary Figure 2: Estimated subject-specific posterior factor trajectories for all patients (untruncated), using the exact method with $q = 50$. The number of latent factors is pre-specified as $k = 2$. For people who did not survive for the full 7-week followup, we plotted their trajectories only before the death date; otherwise trajectories were plotted within a 7-week window after the onset of symptoms.

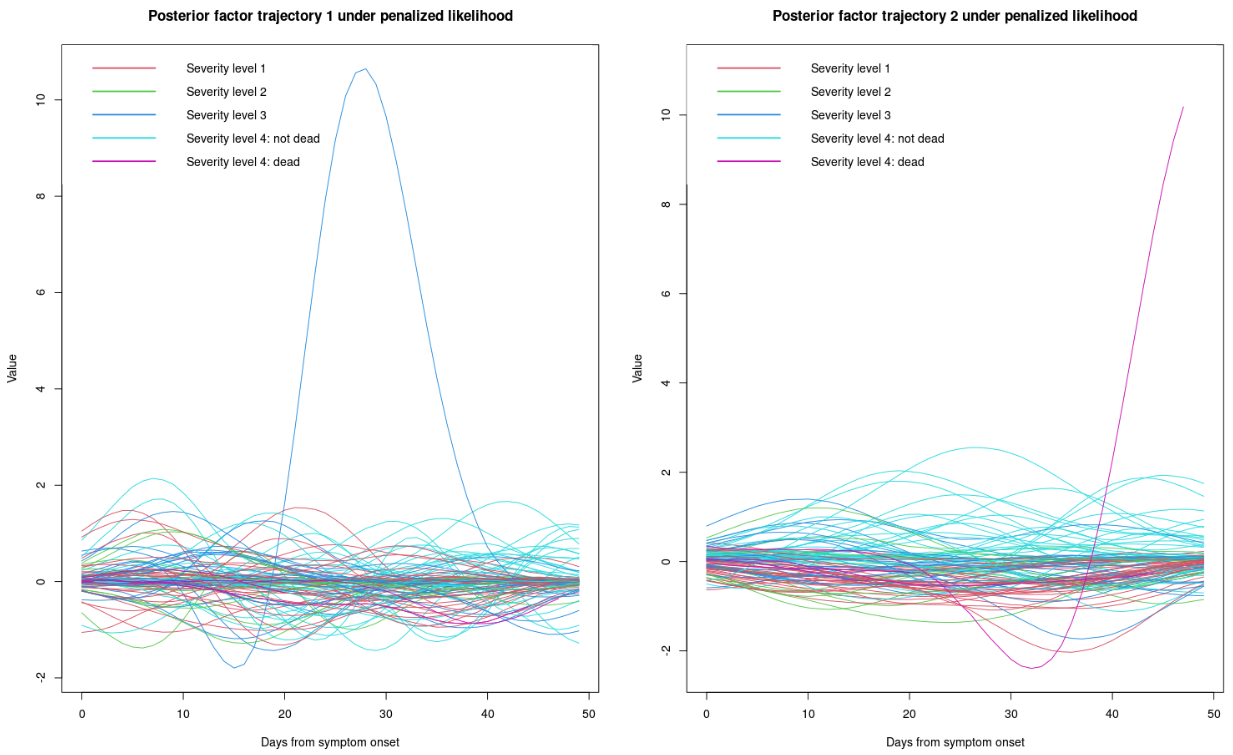

Supplementary Figure 3: Estimated subject-specific posterior factor trajectories for all patients (untruncated), using the approximation method with $q = 8$. The number of latent factors is pre-specified as $k = 2$. For people who did not survive for the full 7-week followup, we plotted their trajectories only before the death date; otherwise trajectories were plotted within a 7-week window after the onset of symptoms.

B.2 Results under the number of latent factors $k = 2$

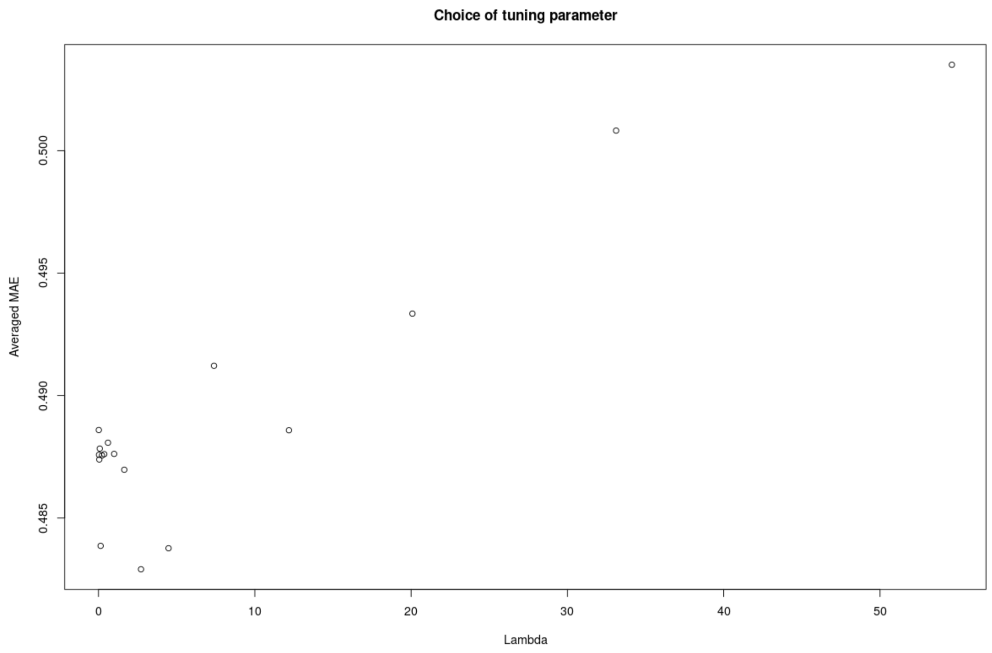

Supplementary Figure 4: Cross validation results for choosing the tuning parameter λ. We vary $\ln(\lambda)$ from -4 to 4, with the length of step as 0.5; this results in 17 candidate values of λ, from 0.02 to 54.60. MAE stands for mean absolute error between estimated biomarker expressions and the truth.

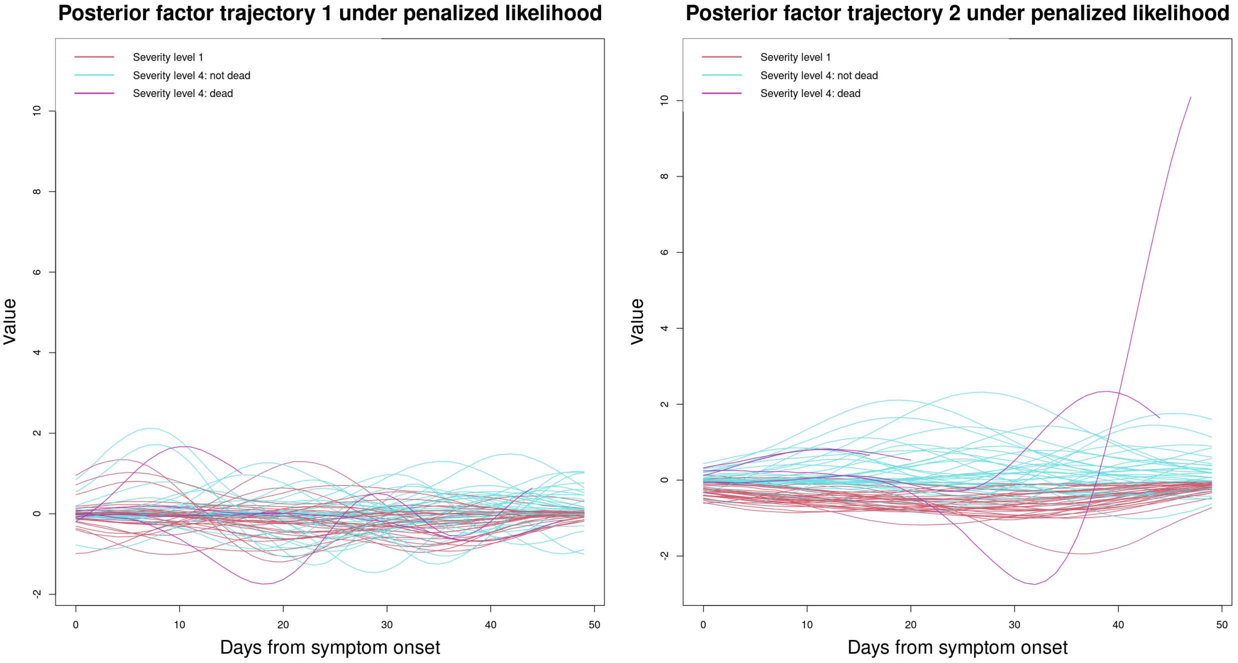

Supplementary Figure 5: Estimated subject-specific posterior factor trajectories for patients with severity levels 1 and 4 only (untruncated), using the exact method with $q = 50$. The number of latent factors is pre-specified as $k = 2$. For people who did not survive for the full 7-week followup, we plotted their trajectories only before the death date; otherwise trajectories were plotted within a 7-week window after the onset of symptoms.

B.3 Results under the number of latent factors $k = 3$

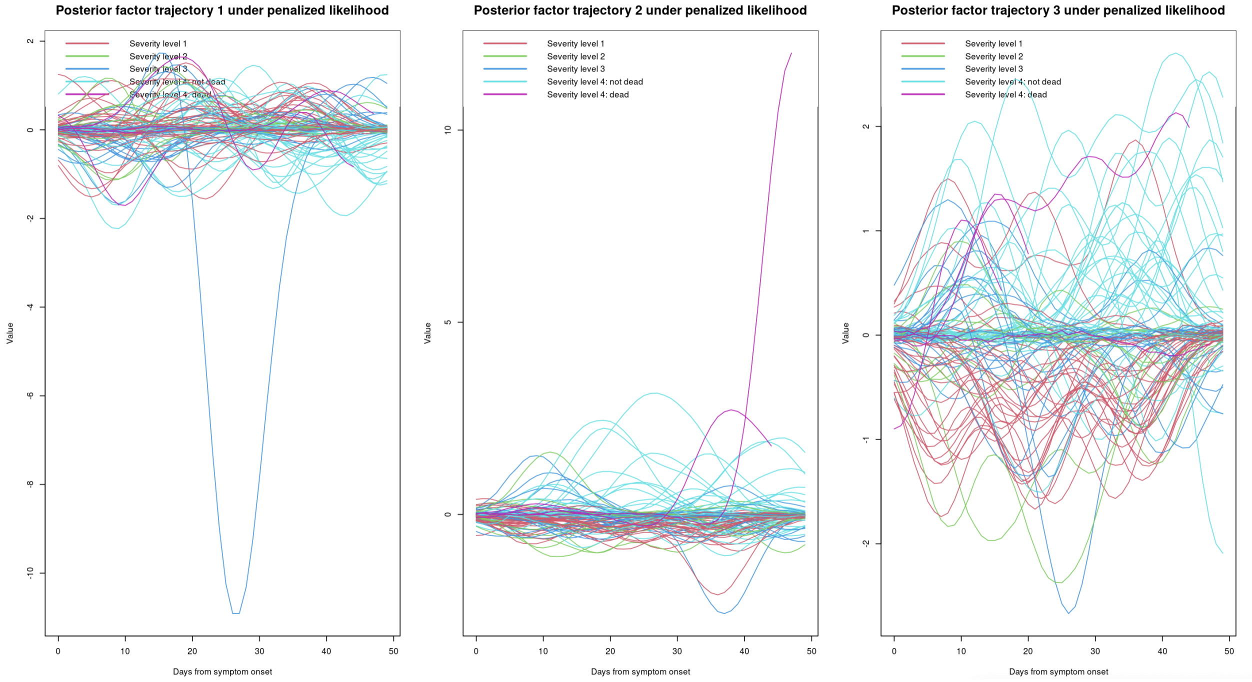

Supplementary Figure 6: Estimated subject-specific posterior factor trajectories for all patients (untruncated), using the exact method with $q = 50$. The number of latent factors is pre-specified as $k = 3$. For people who did not survive for the full 7-week followup, we plotted their trajectories only before the death date; otherwise trajectories were plotted within a 7-week window after the onset of symptoms.

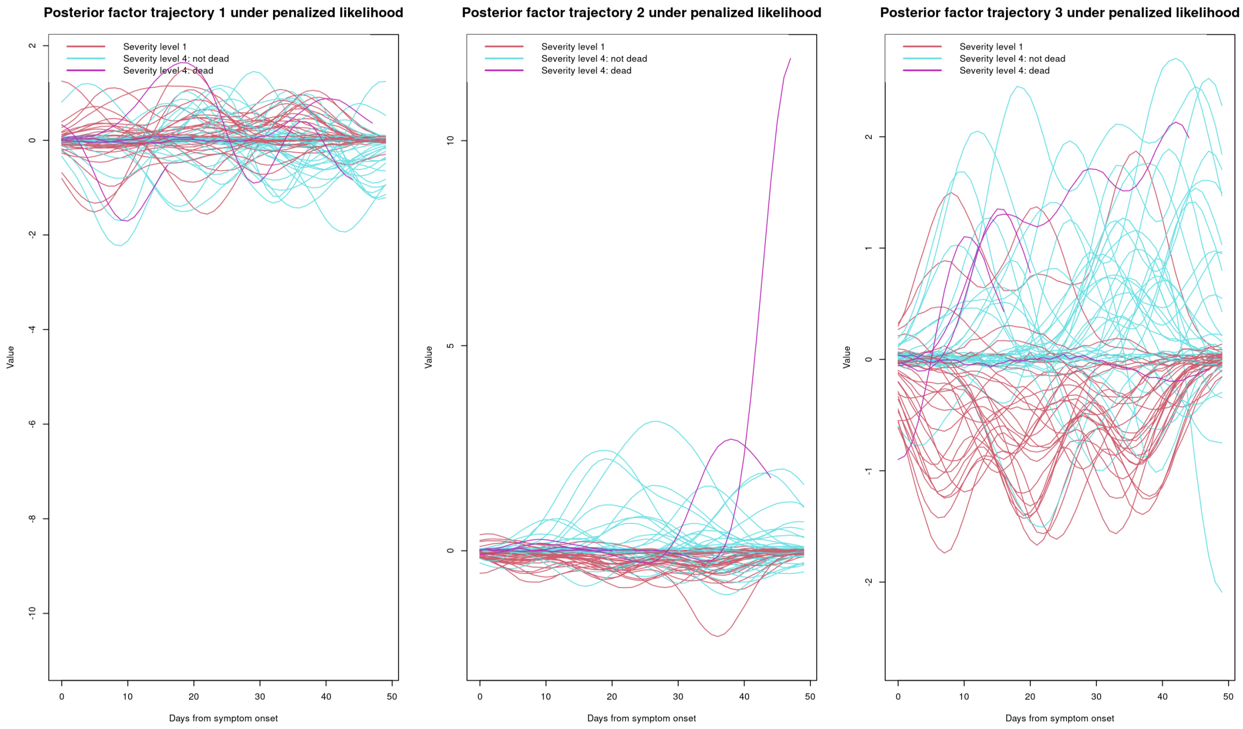

Supplementary Figure 7: Estimated subject-specific posterior factor trajectories for patients with severity levels 1 and 4 only (untruncated), using the exact method with $q = 50$. The number of latent factors is pre-specified as $k = 3$. For people who did not survive for the full 7-week followup, we plotted their trajectories only before the death date; otherwise trajectories were plotted within a 7-week window after the onset of symptoms.

B.4. Standardization of input

When fitting the model to the real-data, we standardized the input of MOGP (i.e., time in our case) so that the range of inputs after standardization matches that in the R package GPFDA. Specifically, we divided the original times (range between 0 and 49) by 49, resulting in the after-transformation times ranging between 0 and 1. When displaying the results, we use the original time for x-axis to facilitate interpretation (i.e., with 'day' as the unit).

Supplementary Table 3: Correspondence between metabolite index and metabolite name.

Metabolite index	Metabolite name
1	3-hydroxyanthranilic acid
2	3-hydroxykynurenine
3	5-hydroxyindoleacetic acid
4	indole-3-acetic acid
5	kynurenic acid
6	kynurenine
7	neopterin
8	picolinic acid
9	quinolinic acid
10	serotonin
11	tryptophan
12	xanthurenic acid
13	1-methylhistidine
15	alpha-aminobutyric acid
16	Arginine
17	Asparagine
18	Citrulline
19	Glutamic acid
20	Glutamine
21	Glycine
22	Histidine
23	Isoleucine
24	Leucine
25	Methionine
26	Phenylalanine
27	Proline
28	Serine

29	Taurine
30	Threonine
31	Tyrosine
32	Aspartic acid
33	Lysine
34	Ornithine
35	Valine

Supplementary Table 4: A summary of the number of observations versus the number of subjects.

Number of observations	Number of subjects
2	50
3	37
4	8
5	6

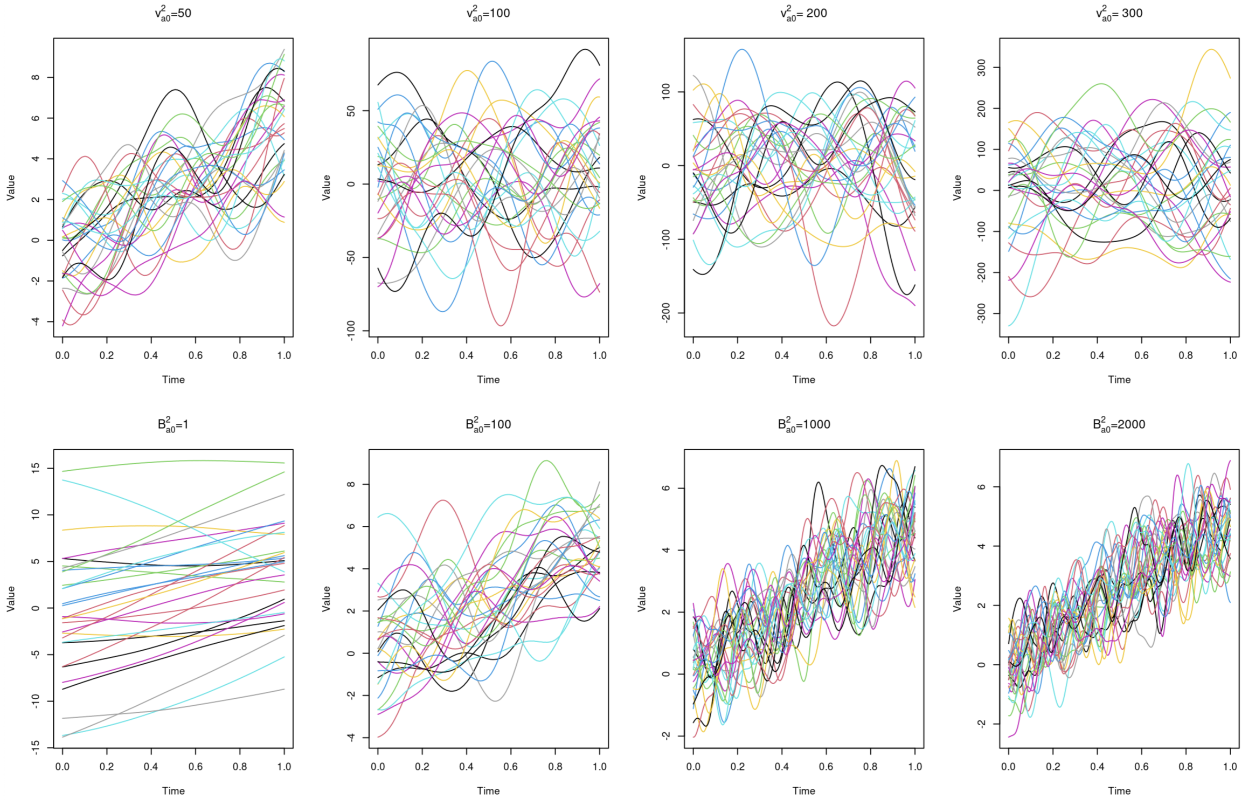

Supplementary Figure 8: 30 sample curves generated from MOGP under different values of v_{a0} and B_{a0}. Note that when varying one hyperparameter, the remaining hyperparameters are fixed.

C. Simulation

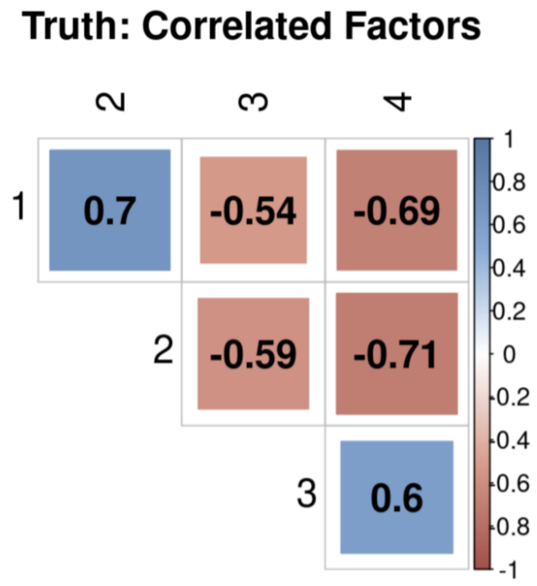

Supplementary Figure 9: True cross-correlations among the 4 latent factors in the simulation study.

References

Ledermann, W. (1937). On the rank of the reduced correlational matrix in multiple-factor analysis. *Psychometrika*, *2*(2), 85–93.

Bekker, P. A., & ten Berge, J. M. (1997). Generic global indentification in factor analysis. *Linear Algebra and its Applications*, *264*, 255–263.

Shi, J. Q., & Choi, T. (2011). *Gaussian Process Regression Analysis for Functional Data*. CRC Press.

Conti, G., Frühwirth-Schnatter, S., Heckman, J. J., & Piatek, R. (2014). Bayesian exploratory factor analysis. *Journal of Econometrics*, *183*(1), 31–57.

Papastamoulis, P., & Ntzoufras, I. (2022). On the identifiability of Bayesian factor analytic models. *Statistics and Computing*, *32*(2), 1–29.
